# Microwave-assisted recycling of tantalum and manganese from end-of-life tantalum capacitors

**DOI:** 10.1038/s41598-025-96574-7

**Published:** 2025-04-11

**Authors:** Ansan Pokharel, Kurundu Shavinka Jayasekera, Edward M. Sabolsky, Terence Musho

**Affiliations:** Department of Mechanical, Materials & Aerospace Engineering, West Virginia University, Morgantown, WV 26505 USA

**Keywords:** E-waste, E-scrap, Carbothermal reduction, Microwave heating, Recycling, Thermodynamics, Mechanical engineering, Environmental sciences, Engineering, Materials science

## Abstract

Critical elements such as tantalum (Ta) and manganese (Mn) are in high demand and subject to supply chain disruptions, underscoring the importance of effective recycling strategies. Tantalum capacitors (TCs), which can contain up to 50% Ta and 18% Mn, represent a significant source of Ta-bearing electronic scrap (e-scrap). Here, we develop a selective carbothermal reduction method driven by 2.45 GHz microwave heating to recover Ta and Mn from end-of-life TCs. Guided by Ellingham and phase diagrams using the CALPHAD approach, the capacitors underwent a three-stage process at varying temperatures and pressures. XRD and ICP-MS analyses confirmed the formation of stable TaC with 97% purity, while Mn was reduced to lower oxide forms. This scalable, selective, and energy-competitive technique offers a new route for the secondary mining of critical metals from heterogeneous e-scrap.

## Introduction

Electronic scrap or waste (e-scrap) refers to discarded electronic equipment or instruments that use electricity^1,2^. Out of the two billion tons of municipal solid waste generated each year^3^, e-scrap contributes about 3 to 5%^4^. E-scrap is the fastest-growing solid waste stream in the world^4–7^, which is of grave concern. In 2024, global e-scrap generation reached an unprecedented volume of 65 million tons^6^. China, the USA, India, Japan, Brazil, Germany, the UK, and Mexico are the top countries generating the most e-scrap in 2024^8^. The total e-scrap generated in each continent/region is shown in Table 1. Despite e-scrap being recognized for containing hazardous products^9^, it is projected to be a multi-billion dollar industry^10^, as it is a potential source for mining rare and costly elements without harming nature when appropriately handled. However, in recent years, only about 17% of e-scrap has been documented to be recycled^11^.

**Fig. 1:**
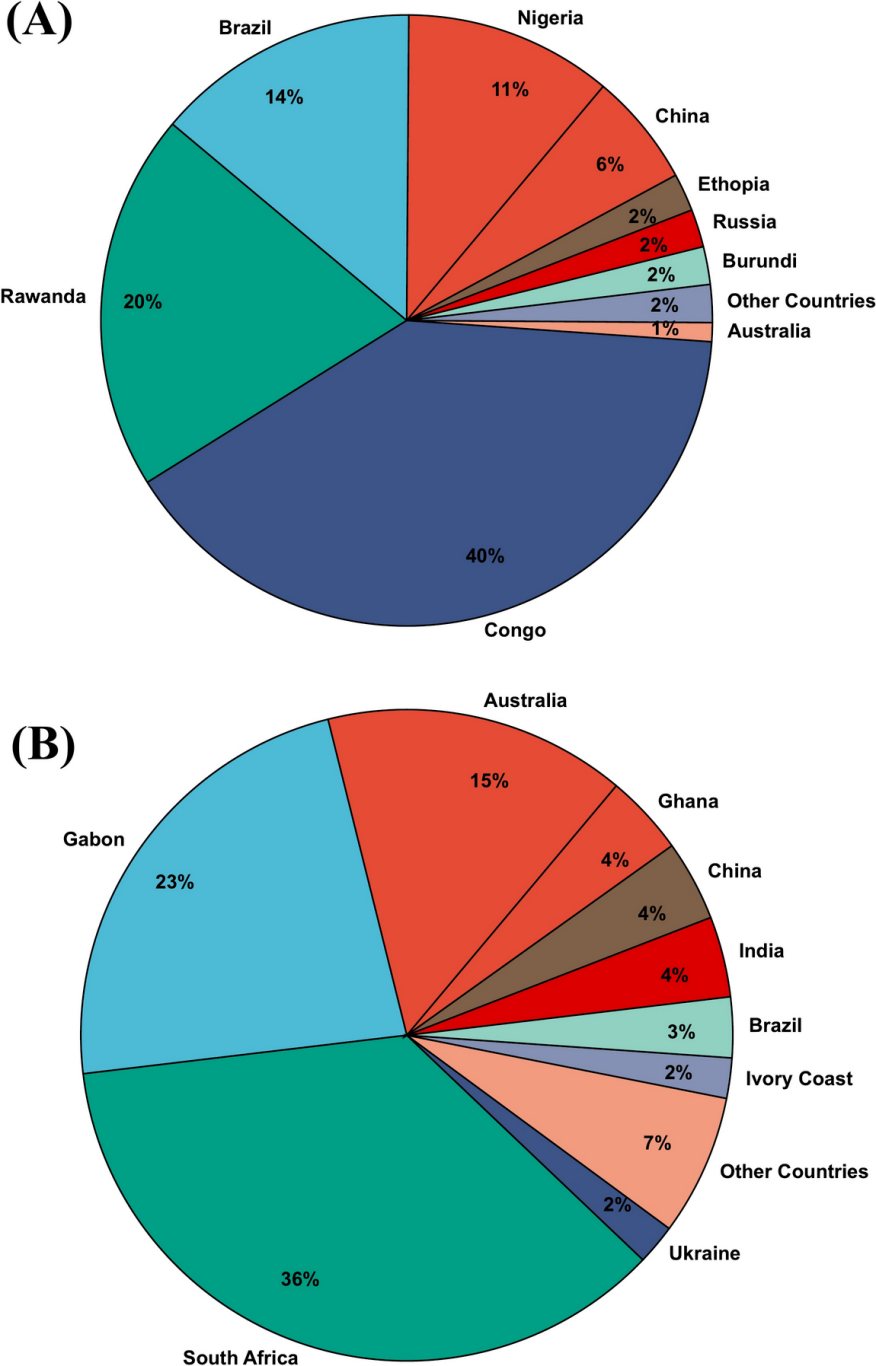
Global ore production data: (**A**) Global Ta ore production based on 2023 USGS Mineral Commodity Summaries^25^ and (**B**) Global geographic distribution of Mn ore production in 2023^30^.

E-scrap is often categorized as a secondary mining source of expensive and rare elements like tantalum, manganese, gold, indium, gallium, platinum, silver, neodymium, dysprosium, and many others^12,13^. One of the main tantalum-rich components in e-scrap is surface mount capacitors. Almost every modern electronic and electric device, especially portable and compact ones such as mobile phones, laptops, and notebooks, contains tantalum capacitors (TCs). TCs have a high capacitance-to-volume ratio, high energy density, high oxidation resistance, and thermal stability^14,15^, attributed to the favorable dielectric properties and high operating temperature of tantalum (Ta). Each TC contains about 30% to 50%^16–18^ Ta by weight, varying by capacitance and manufacturer. The demand for TCs is growing annually, as shown in Fig. 1B, primarily driven by their increasing use in modern electronics^19,20^. Recently, new consumer markets, such as those for 5G/6G networks and electric vehicles, have further driven the demand for Ta^21^. Therefore, it is reasonable to speculate that the demand for Ta will surpass the projected production of general e-scrap.

Ta has been classified as a critical raw material by the European Commission^22^ and the United States^23^, with a Clark value of only 0.0002 wt%^24^. The major production of Ta comes from Central African countries, mainly Rwanda and the Democratic Republic of the Congo (DRC), contributing more than 50% of the global demand. The annual Ta production in various nations is shown in Fig. 1A. However, measures taken by these countries have led to a 26% decline in Ta production in 2020^25^, disrupting the supply chain and creating urgency in the global market. In addition to Ta, manganese (Mn) is the second-largest element in modern TCs, comprising about 18%^15,26^. It is the fifth most used metal after iron, aluminum, copper, and magnesium, with an annual ore production of 20.3 million tons in 2020^27^. The global Mn ore production of 2018 is shown in Fig. 1B. The major sources of Mn are in South Africa (74%) and Ukraine (10%)^28^. Recently, Mn has been categorized as a critical element due to ore depletion and supply chain disruptions post-COVID. With the compound annual growth rate (CAGR) of TCs projected to be about 6% over the next decade^29^, it is vital to implement cradle-to-grave recycling for TCs and seek efficient approaches to recycle and extract Ta and Mn, rather than relying on a few production sources.

**Table 1:** Global e-scrap generation in 2024 for select geographic regions, according to the global e-waste monitor (GEM)^6,8^.

Continent/region	Total E-scrap generated (Mton)	E-scrap per capita (kg/year)
Africa	3.79	2.5
Americas	8.65	14.1
Asia	30.76	6.4
Europe	13.10	17.6
Oceania	0.74	16.1

Europe and Oceania regions have thehighest per capita generation of e-scrap, followed closely by the Americas.

### Microwave heating of materials

Microwave heating (MWH) is a form of direct volumetric heating of materials using electromagnetic waves (EW) in the microwave frequency range. An EM wave within the frequency range of 1 GHz to 1000 GHz, corresponding to wavelengths from 30 to 0.03 cm, is termed a microwave (MW)^31^. The development of MW technology accelerated post-World War II with the invention of microwave radar for detecting enemy aircraft^32^, although the term “microwave” was initially coined by A.G. Clavier in 1931^33^.

Microwaves are generated using various devices, including magnetrons, power grid tubes, traveling wave tubes, cross-field amplifiers, klystrons, and gyrotrons. Their applications are diverse, ranging from communication^34^ to food heating in household kitchens^35,36^. Like other electromagnetic waves, propagating microwaves consist of perpendicular electric and magnetic fields oscillating in phase within a plane perpendicular to their direction of propagation, making them transverse waves. The speed of microwave propagation, or phase velocity, varies depending on the medium; in a vacuum, microwaves travel at the speed of light without attenuation. The depth of penetration and power loss within a medium depends on its complex index of refraction or complex dielectric properties. While microwaves have very shallow penetration depths in free-electron conductors such as metals, their penetration depth is much larger in semi-transparent dielectric insulators, allowing partial transmission with attenuation of intensity^37^. Microwave interactions with materials are typically categorized into three types: opaque materials, which absorb or reflect microwave fields; semi-transparent materials, which attenuate the signal while permitting partial transmission; and transparent materials, which allow microwaves to pass through with minimal or no attenuation.

As a propagating electric field traverses a material, the intensity of the EM wave attenuates due to the formation of electronic and magnetic dipoles^38^. The fundamental mechanism responsible for material heating is related to the relaxation of these dipoles. In the context of electric dipoles, a dipole moment arises when the centers of positive and negative charges in a material do not coincide. Mathematically, the dipole moment is given by, $\vec {p} = q \vec {d}$, where $\vec {p}$ is the dipole moment, *q* is the charge, and $\vec {d}$ is the distance between the two opposite charge regions. These dipoles can originate due to different mechanisms and are classified as (i) electronic polarization and ionic polarization, (ii) orientational polarization, and (iii) interfacial polarization^39^. In the first case, when an atom is exposed to the electric field, the electron cloud surrounding the positive nucleus gets distorted, separating the center of the negative and positive charge. The dipole moment induced by the displacement of the center of charge is defined as electronic polarization or optical polarization. When ionic materials or polyatomic molecules are placed in the electric field, the positive and negative ions get displaced relative to each other. This phenomenon is defined as ionic polarization or vibrational polarization as it results in the direct distortion of the lattice and is directly coupled with the normal lattice vibrations. At the molecular level, for lone molecules like water, there exists an apparent permanent dipole due to charge distribution, even in the absence of the electric field. When an electric field is applied to these materials, the dipole gets oriented in the direction of the electric field, resulting in orientational polarization or dipolar polarization. Similar to ionic polarization, because there is reorientation between the built-in polarization and the resulting orientational polarization, there is coupling to the lattice vibrations of the molecule. A final type of electric polarization arises at interfaces in the form of interfacial or space charge polarization. This is induced when the dipole charges arise at the interface of the material due to a partial bonding state^39,40^. Electron-phonon coupling at the interface will induce heating of the interface as the interfacial dipoles relax. Each of these polarization mechanisms has a resulting difference in resonance frequency and corresponding relaxation time. The atomic electronic polarization occurs at a resonance frequency slightly above $10^{14}$ Hz. The amplitude or intensity of the coupling will increase when the frequency of the forcing function (EM wave frequency) is near the resonance frequency. That being understood, an off-resonance forcing function can still achieve quantifiable coupling or heat generation. The dipole relaxation time can be correlated to the probability of a scattering event at the associated length and time scale. Typically, ionic polarization is induced at the frequency range of $10^{10}$ Hz to $10^{13}$ Hz, optical-type EM waves. The orientational polarization is induced at the frequency around the MW range. The interfacial polarization occurs at a relatively low-frequency range of 10 Hz to 10 kHz^41^. For this research, the main mechanism of heat generation is the relaxation of dipoles due to the electronic polarization and ionic polarization within an opaque or semi-transparent dielectric material. It should be noted that a similar argument can be deduced for magnetic dipoles, but (1) the field strength of the magnetic field is significantly lower than the electric field strength in this application and (2) a material must possess attributes of microscopic magnetism (unpaired electrons with nonzero spin) or macroscopic magnetism (collective alignment of spins) to form magnetic dipoles.

The total polarization in a material is defined by polarization density ($\vec {P}$), which is the sum of the dipole moment over the volume of the sample. Polarization density qualitatively defines the polarization as shown in Eq. 1. It is directly proportional to the applied electric intensity $\vec {E}$. The $\varepsilon _0$ and $\chi$ are the permittivity of the free space and dielectric susceptibility, respectively. The dielectric susceptibility represents the degree to which the material polarizes, i.e., the greater the value of susceptibility, the higher the material gets polarized. This infers that the value at vacuum is zero. Along with $\vec {P}$, electric flux density ($\vec {D}$) or electric displacement is another vital term used in understanding the dielectric property of the material. It represents the total charge per unit area that would be displaced in a material when placed in an electric field. The relation between $\vec {P}$ and $\vec {D}$ is shown in Eq. 2. The electric displacement is also proportional to the applied electric field (Eq. 3). Hence, the mathematical relation between $\varepsilon$ and $\chi$ can be represented as shown in Eq. 4. Since the value of $\chi$ can never be below zero, $\varepsilon$ is always greater than or equal to one.

The fundamental difference between these two constants is that $\chi$, measures the degree of polarization while $\varepsilon$ quantifies the charge-storing capacity of the material as the capacitance is directly proportional to the permittivity of the dielectric materials.

1 $$\begin{aligned} \vec {P} = \varepsilon _0 \chi \vec {E} \end{aligned}$$

2 $$\begin{aligned} \vec {D} = \varepsilon _0 \vec {E} + \vec {P} \end{aligned}$$

3 $$\begin{aligned} \vec {D} = \varepsilon _0 \varepsilon \vec {E} \end{aligned}$$

4 $$\begin{aligned} \varepsilon = 1 + \chi \end{aligned}$$

The complex permittivity quantifies the material’s dipole response and relaxation behavior in the oscillating EM field. Mathematically the complex dielectric constant $\epsilon$ comprises a real number component ($\epsilon '$) and the imaginary number component ($\epsilon ''$). The relationship between the dielectric constant and the real and imaginary quantities takes the following form,

5 $$\begin{aligned} \epsilon = \epsilon _r \epsilon _0 = \epsilon ' - i \epsilon '', \end{aligned}$$

where $\epsilon _r$ is the relative permittivity.

The loss tangent, which is a common quantity used to quantify the dipole relaxation of a material due to microwave excitation is described by the following expression,

6 $$\begin{aligned} \tan {\delta _e} = \epsilon '' / \epsilon ' = \frac{\omega \epsilon '' + \sigma _e}{\omega _e \epsilon '}, \end{aligned}$$

where $\sigma _e$ is the frequency-dependent electrical conductivity and is assumed to be zero for electrical insulators and most dielectrics. For free electron conductors $\sigma _e=const=\sigma _{dc}$, which will be significantly influenced by temperature. For all materials the higher the loss tangent, the more readily the material can be heated.

The energy or electrical potential storage capacity of a material in an electric field is represented by the real part ($\epsilon '$) while the complex part ($\epsilon ''$) represents the energy dissipation capacity of the material from the electric field to heat energy^42^. The ratio of the imaginary part and the real part is referred to as the tangent loss and can be represented as shown in Eq. 6^33^. The loss tangent can be related to the inverse of the quality factor ($\tan {\delta _e} \propto 1/Q$) of a simple oscillator. Similar quantities can be written for magnetic dipoles, called the permeability, as seen in the following complex equation,

7 $$\begin{aligned} \mu = \mu _r \mu _0 = \mu ' - i \mu ''. \end{aligned}$$

Similarly, the real part of the permeability quantifies the energy storage behavior. The permittivity can be thought of as a capacitive term, whereas the permeability can be thought of as an inductive term, both storing energy. The imaginary part ($\mu ''$) again quantifies the loss or relaxation of the magnetic dipoles. It should be noted that a material that is nonmagnetic will have a $\mu$ of unity, as in the case of the materials for this study.

Since $\epsilon ''$ and $\mu ''$ quantify the loss due to dipole relaxation under the influence of the electric and magnetic fields^36^ they are critical to quantifying the microwave heating effect. When the EM field oscillates, the material cannot follow the rapid change or the reversal of the field. Due to this, the polarization lags the field, and hence the heating gets induced in the material^36,43^. The total heat generated in a material is thus the combination of the electric and magnetic losses. The time-averaged total power loss ($Q_H$) for a dielectric material is shown in Eq. 8. The total loss term will have units of power per unit volume and can be equated to the volumetric heat generation within a material. The $E_{rms}$ and $H_{rms}$ are the root mean square components of the electric and magnetic fields respectively and $\omega$ is the angular frequency ($\omega =2 \pi f$) of the incident field. This equation illustrates that the volumetric heating can be increased by increasing the imaginary components of the dielectric properties by increasing the magnitude of the electric field (*E*) or the magnetic field strength (*H*), or by increasing the frequency of the incident field.

8 $$\begin{aligned} Q_H = \omega \epsilon '' {E^2_{rms}} + \omega \mu '' {H^2_{rms}} \end{aligned}$$

In the conventional heating process, the modes of heat transfer from the heat source to the material are surface conduction, convection, and radiation. The heat transfer rate from the source to the material is usually much lower when compared to MW heating because of physical material limitations (e.g. thermal conductivity of oxides). As a result of the material property limitations the depth of penetration of heat at earlier times during conventional heating^44^ is much shallower when compared to microwave heating. MW has a higher penetration depth as a result of being able to generate heat over a much larger region of the sample. MW is no longer limited by a surface effect but relies on a volumetric effect. MW heating works on the principle of direct energy conversion of electromagnetic energy to thermal energy. Further, MW heating provides the advantage of selective heating when the sample is a mixture of various compounds^45^ as a result of the materials’ dielectric properties. MW heating is fast, efficient, can be turned on and off quickly, and is not limited by surface properties^46–49^.

### Microwave-driven carbothermal reduction

Carbothermal reduction (CTR) is the chemical process of reducing a precursor compound (typically a metallic oxide) by mixing it with carbon (C) and heating it in an inert atmosphere^50^. CTR is an endothermic reaction that usually occurs at high temperatures^51^. The general form of CTR is shown in Eq. 9, where $a$ and $b$ are integers based on the stoichiometry of the oxide, and $M$ represents the metal. The complete carbothermal reaction yields carbon monoxide (CO) or carbon dioxide ($\hbox {CO}_{2}$) and a metal, whereas the incomplete reaction generates lower forms of the metallic oxides or metal carbide and CO^52^. The relative formation of CO versus $\hbox {CO}_{2}$ primarily depends on the temperature and the partial pressure of oxygen present.

9 $$\begin{aligned} {\rm M}_a{\rm O}_b + bC \rightarrow b{\rm CO} + a{\rm M} \end{aligned}$$

For this research, the reduction method is based on an extractive metallurgy process that purifies the metal by selectively reducing the oxide to its metallic form. This process is akin to basic steelmaking, in which iron ore or iron oxide is reduced to pig iron. However, there are several key differences. One difference is that the resulting materials are physically separated as a metal sponge in the solid phase after reduction. In steelmaking, the physical separation occurs in the molten state that flows out of the bottom of the reactor. Moreover, steelmaking relies on the injection of air, which reacts with carbon or coal to produce exothermic energy that drives the process. In the present process, although some energy is derived from the exothermic reaction of carbon with the oxide, most of the energy is supplied by the microwave source. This approach also stabilizes the metal by controlling the partial pressure (primarily of oxygen). Additionally, no flux additions are required beyond the ash present in the capacitors because the electronic scrap, once ground to micron-size particles, is already phase-separated. The resulting purity, as will be discussed, is similar to that of a typical pyrometallurgical process, such as basic steelmaking.

The reduction of refractory metals such as Ta, when performed using conventional thermal heating (CTH), requires high input power due to the high reduction temperature and the long reaction time^46^. Additionally, the heat rate in conventional CTR is considerably slower, which can result in cold centers in pelletized samples, especially at early time^44^. The first documented application of microwave energy in metallurgical material treatment processes dates to the mid-1960s^53^. However, the specific application of microwave heating (MWH) for reducing metal oxides began in the 1990s^45^. Unlike CTH, where heat is transferred to the sample through surface heating, MWH works on the principle of volumetric heating. The rate of heating for MWH becomes a balance of volumetric and surface heat transfer effects. Furthermore, MWH offers the advantage of selective heating in mixtures of various compounds^45^. Overall, MWH is faster, more efficient, and cleaner, and it can produce results comparable to, or better than those obtained using conventional methods, all while requiring less input power^46–48^.

To illustrate the effect of MWH, consider the reduction of zinc oxide and zinc ferrite, which under conventional thermal heating requires temperatures of between 850$^\circ$C and 950$^\circ$C, respectively. In contrast, microwave heating can lower these temperatures to 550$^\circ$C and 450$^\circ$C^54^. In a study by Huang et al.^55^, who examined the reduction of iron oxide to iron using both carbothermal reduction and MWH, the latter significantly decreased the activation energy of the overall process. This decrease was attributed to the rapid, volumetric heating caused by the interaction between the microwave field and the material, effectively lowering the reduction temperature.

Similarly, Lee et al.^56^ conducted a thermodynamic assessment of Gd-doped ceria oxide ($\hbox {CeO}_{2}$) to investigate the influence of MWH on metal oxide reduction. Using the Van’t Hoff method, they found that the reduction enthalpy in the presence of microwaves was nearly half that of conventional thermal reduction. Their further analysis, based on defect equilibria models, showed that MWH lowers activation energy, accelerates defect formation, and reduces reaction time. This study, which integrates real-time oxygen monitoring, defect modeling, and thermodynamic analysis, provides new insights into the role of microwaves in metal oxide reduction. MWH has also been shown to enhance material densification, leading to smaller grain sizes^57^ and greater microstructural uniformity of the material^58^.

Several studies have examined the carbothermal reduction of Ta and Mn oxides using CTH^59–61^, and extensive work has been conducted on elements such as Fe, Ni, Co, Cu, Pb, Zn, Al, Mg, Ti, Au, Ag, and Pt under MWH-based CTR^45,62^. However, relatively few investigations have focused specifically on producing Ta and Mn using MWH. Chen et al.^63^ conducted a microthermal-enhanced selective carbothermal reduction of low-grade pyrolusite to manganese oxide (MnO), demonstrating that MWH lowered both the roasting temperature and time. In a comparative study, He et al.^64^ found that CTR of Mn ore was more rapid under MWH due to “hot cracking,” which increases the reaction surface area, as well as the strong interaction between C and microwaves that enhances gasification. Hassine et al.^46^ likewise observed that microwave-assisted carbothermal reduction of $\hbox {Ta}_{2}\hbox {O}_{5}$ to TaC proceeded much faster than under CTH. The reduction temperature and reaction time were 1500$^\circ$C and 60 min, respectively, compared to 1750$^\circ$C and 6 hours with CTH.

Previous studies have also explored MWH for extracting metals from ores, lower oxide forms, or metal carbides derived from single-compound sources. In a recent publication, Ahmed^65^ employed MWH-based CTR on low-grade Egyptian Mn ore. Because the ore contained iron oxide, selective reduction yielded 97% pure iron, while the manganese oxide was reduced to Mn at 84.45% purity. No other studies, however, appear in the literature describing the separation of multiple metals from a complex mixture using MWH, except for Ahmed’s work^65^. This study focuses on exploiting the selective heating phenomenon of MWH to extract Ta and Mn from a mixture of samples prepared from tantalum-capacitor e-scrap. Because the optimal reduction temperature and pressure differ for each composition, staging the carbothermal reduction process to provide selective separation required precise control of the operational conditions to achieve selectivity.

**Fig. 2:**
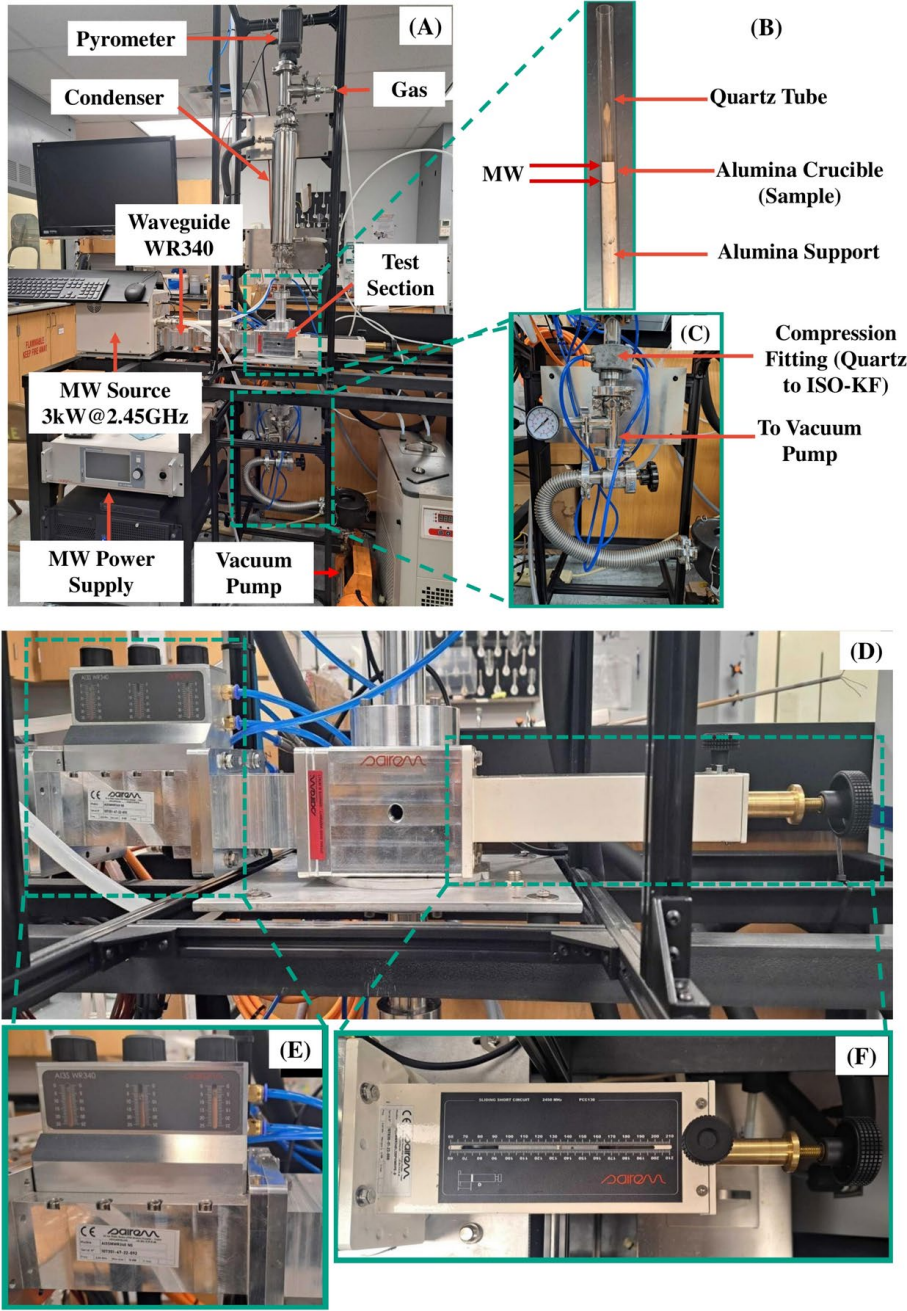
Photograph of the experimental microwave test bed. (**A**) Overview of the test bed components, showing the microwave source and horizontally positioned waveguide. (**B**) Close-up of the internal reactor section, illustrating the sample placement within the reactor. (**C**) A vacuum pump used to evacuate the reactor and maintain the desired partial pressure. (**D**) Close-up of the waveguide section featuring a (**E**) three-stub tuner and a (**F**) sliding short. (**E**) and (**D**) were used to match the load (sample’s) impedance to provide an efficient transfer of power through the waveguide. The initial positions of the stub tuner and sliding short were determined using COMSOL Multiphysics software and subsequently fine-tuned empirically.

## Methods

### Experimental setup

The experimental testbed used in this study is shown in Fig. 2. The setup features a single-mode microwave (MW) generator (Sairem Microwave and Radio Frequency, Décines-Charpieu, France), which uses a 3 kW, 2.45 GHz magnetron-based source. The test section is a water-cooled aluminum applicator with a WR-340 waveguide attachment. A quartz tube is inserted vertically through the applicator in the direction of the electric field with respect to TE01 waveguide mode. The space between the quartz tube and the WR-340 waveguide is air-blasted to facilitate convective cooling of the outer surface of the quartz tube.

The sample was placed inside an alumina crucible (Fig. 2B), supported from below by an alumina (Al_2_O_3_) tube. The alumina tube has a fixed height designed to position the crucible at the center of the waveguide within the applicator test section. Both the alumina crucible and alumina tube were housed within a quartz tube. The lower and upper ends of the quartz tube were connected to a vacuum fitting (Fig. 2C) via a water-cooled compression fitting, which transitions the quartz tube to a KF-style vacuum connection. The lower KF connection was connected to a vacuum pump with a vacuum trap.

A short-wavelength (900 nm Williamson IR) pyrometer (not shown in Fig. 2) was used for remote temperature monitoring through a coated quartz demountable viewport. The pyrometer, mounted above the sample, measured the top surface of the sample within an integration diameter of about 5 mm. Its emissivity was set to 0.95, corresponding to carbon black.

Initially, the vacuum system was drawn down to − 722 Torr gauge (− 28.4 in-Hg, or 95% vacuum). It was then backfilled with argon from a port near the pyrometer until atmospheric pressure (0% vacuum) was reached. Next, the system was evacuated again to − 722 Torr gauge and maintained at this pressure for the remainder of the experimental runs. This vacuum environment served two purposes: (1) controlling the partial pressure of oxygen and (2) initiating and sustaining plasma around the sample.

A cylindrical condenser (heat exchanger) was mounted above the reactor (Fig. 2) to serve two functions. The condenser employs a concentric-tube design in which the outer region carries a water/glycol mixture at a constant temperature, maintained by a chiller, while the inner tube is a smooth cylinder of constant diameter where the gases are exposed. As the reaction proceeds, the condenser helps cool the evolving chamber gases (primarily Ar, CO, $\hbox {CO}_{{2}}$) and condenses any volatile species liberated in the plasma.

The temperature during experiments was controlled via a custom LabVIEW (Laboratory Virtual Instrument Engineering Workbench) program implementing a proportional-integral-derivative (PID) controller. The desired heating profile was defined by a ramp rate and one or more dwell temperatures entered into the software based on the target product phases. During operation, the pyrometer continuously measured the sample temperature in real-time, with the PID controller adjusting the microwave power. The PID controller was tuned using LabVIEW’s autotuning feature to characterize the system response. Microwave power was managed through a Modbus Ethernet interface connected to the MW power supply, and pyrometer readings were received by LabVIEW over a USB serial connection.

### Setup optimization

The microwave (MW) system shown in Fig. 2D consists of a manual stub tuner (Fig. 2E) and a sliding short (Fig. 2C), both used to tune the standing wave in the single-mode system. The efficiency of the heating process depends on matching the waveguide’s impedance with that of the load, which in this system is done by adjusting the lengths of the stubs and the sliding short. As the stubs are extended into the waveguide, they vary the inductance in the waveguide and thus change the impedance, with the goal of achieving an optimal match and minimizing reflected power. The sliding short, on the other hand, can be used to adjust the phase (or position) of the peak electric field within the sample. However, altering the sliding short also changes the waveguide length, thereby affecting the impedance. Consequently, both the stub tuner and sliding short must be optimized.

One practical way to find their optimal positions is to monitor the reflected power while simultaneously adjusting both components. Although this approach can be successful, it may not always reach the theoretical optimum. To address this, we conducted a high-frequency RF finite element simulation in COMSOL Multiphysics version 6.1 (see Supplemental Information for model details) to determine the positions of the sliding short and tuning stubs that would maximize power transfer into the sample. The simulation incorporated the entire geometry of the test bed and the temperature-dependent dielectric properties of the sample (Fig. S1), as well as the three stubs and the sliding short. The model outputs provided initial positions for these components, which were then validated experimentally; only slight adjustments were necessary. Additionally, the COMSOL results provided an estimate of the peak heating rates and temperatures achievable with our reactor setup (Fig. S2).

### Sample preparation and characterization

The sample preparation procedure in this study was carefully designed to ensure consistency and reproducibility. Tantalum capacitors (TCs) (KEMET Tantalum Capacitors PCB 10 $\upmu$F SMD, 10% tolerance, 10 VDC, Case Size 3216, T491 Series) extracted from end-of-life electronics (containing approximately 42% Ta) were selected as the primary source material. To concentrate the elemental components and prepare the material for further processing, the capacitors were pyrolyzed in air at 850$^\circ$C for four hours in a tube furnace, then allowed to cool to room temperature. Pyrolysis was required to convert the plastic casings, binders, paints, and pigments on the TCs into carbon. After pyrolysis, the capacitors were more easily shredded and finely crushed using a mortar and pestle, ensuring sample homogeneity and consistent microwave carbothermal reduction.

Next, 1 *g* of the pyrolyzed TCs was combined with 0.2 g of carbon black (lampblack) powder (lot no. S22E047, Alfa Aesar 39724, Thermo Fisher Scientific). The added carbon black increased the overall carbon content to about 40% above the stoichiometric requirement for carbon relative to tantalum pentaoxide ($\hbox {Ta}_{2}\hbox {O}_{5}$). Sufficient carbon was needed to facilitate effective microwave heating, as carbon serves as a strong microwave absorber. Moreover, oversaturating the mixture with carbon does not significantly alter the target phases to be recovered, as confirmed by calculated phase diagrams.

The pyrolyzed TC material was characterized using inductively coupled plasma mass spectrometry (ICP-MS, NexION 2000 by PerkinElmer). The resulting elemental composition, excluding carbon, is provided in Table 2. The pyrolysis process was found to enrich the sample in metallic constituents relative to the original mixture because the mass of organics (plastics and paints) were lost during pyrolysis.

**Table 2:** ICP-MS data for capacitors after pyrolysis and before microwave processing. The reported percentages do not include the carbon content.

Elements	Composition (%,[ppm])
Tantalum	78.486 [78,486]
Manganese	20.598 [20,598]
Nickel	0.9157 [9,157]

**Fig. 3:**
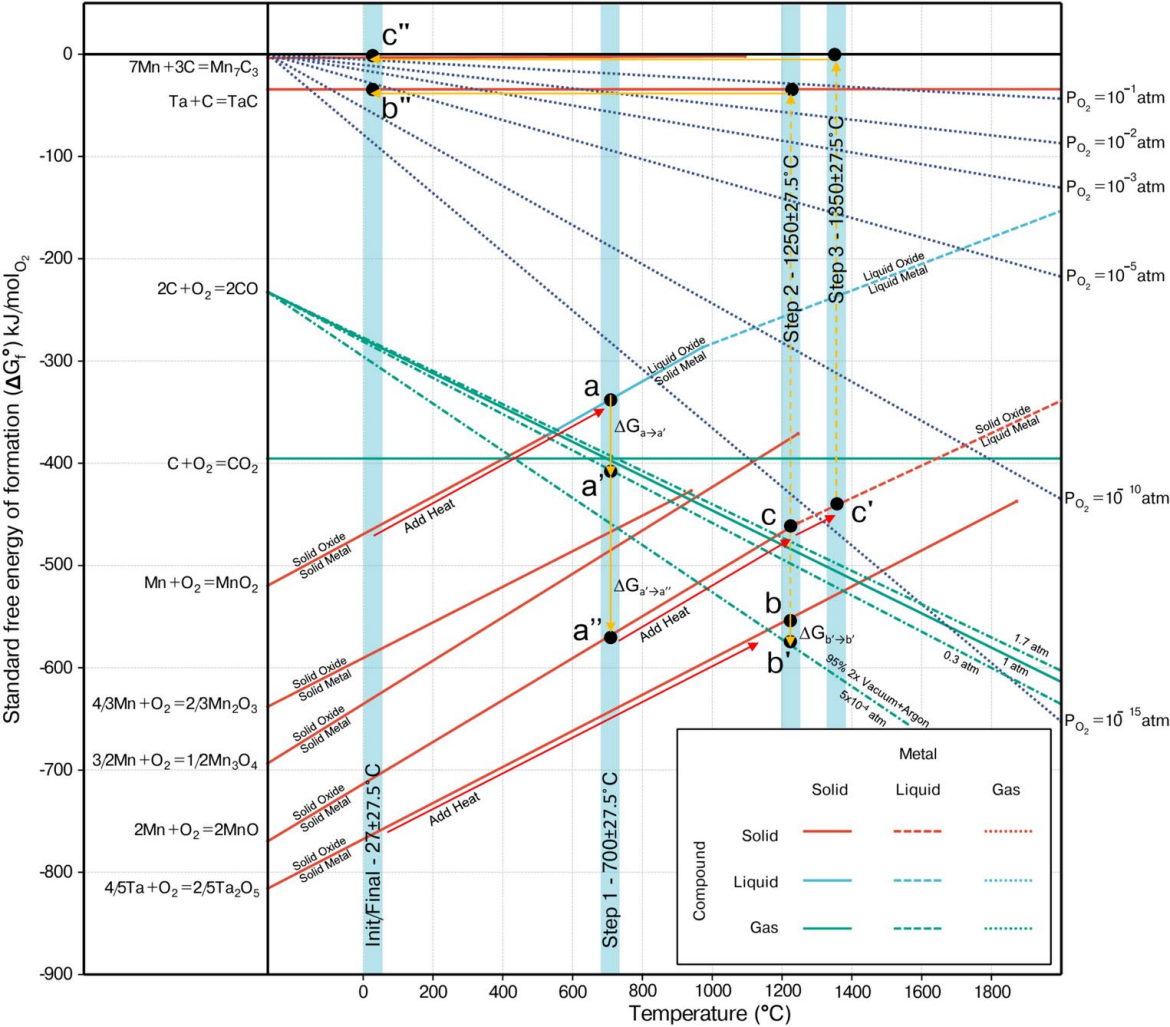
Ellingham diagram for the oxides and carbides of Ta and Mn, illustrating the temperatures at which reduction becomes thermodynamically favorable ($\Delta$G$_{1\rightarrow 2}<$0). Three processing steps were employed to refine the TCs in this study, with the ED serving as the initial reference for process control.

### Determination of temperature and pressure for microwave processing

**Fig. 4:**
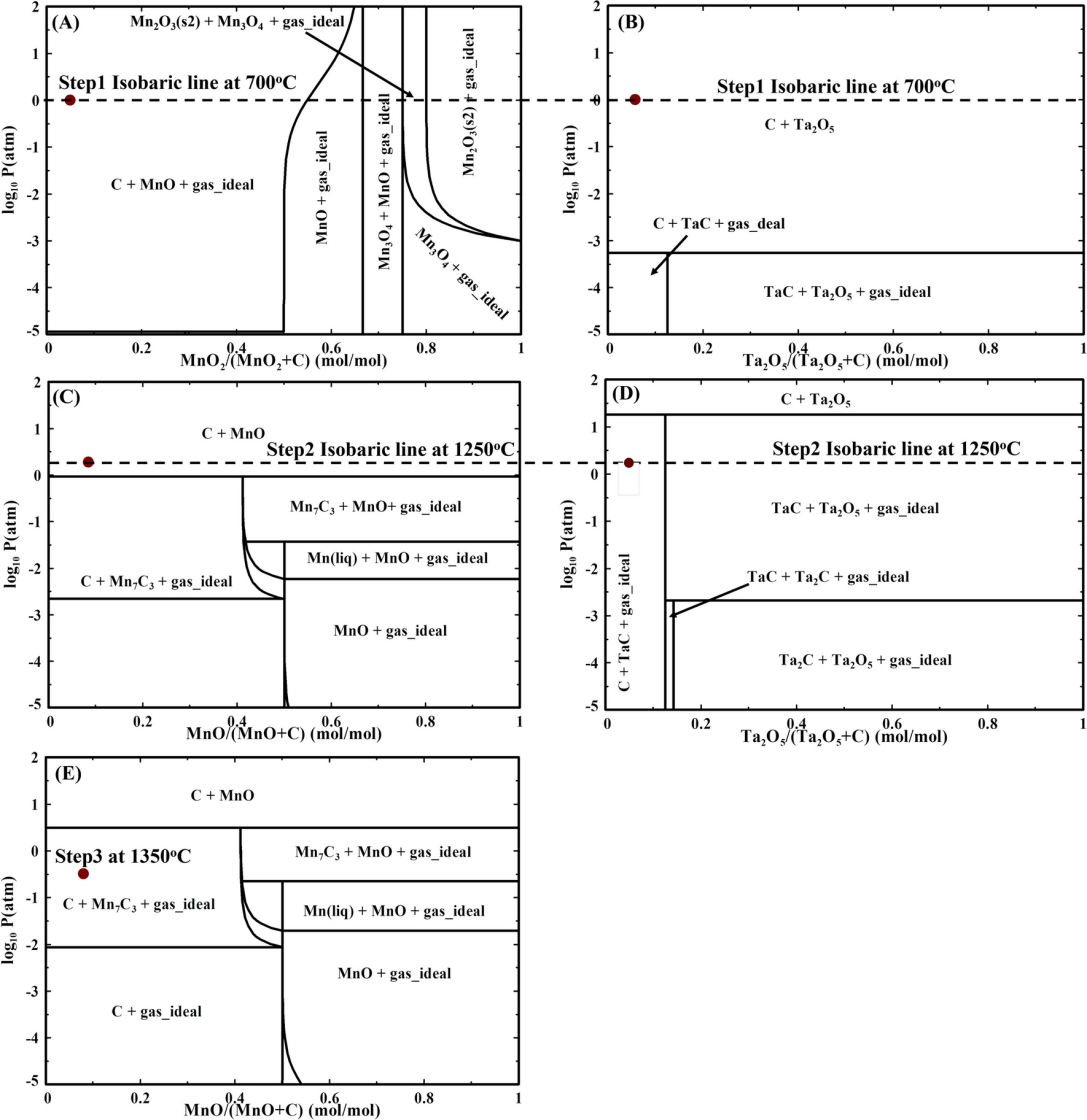
Phase diagram showing the pressure for each reduction step. In (**A**) and (**B**) the red dot indicates the reduction pressure for Step 1, with the temperature maintained at 700$^{\circ }$C. During Step 1, $\hbox {MnO}_{2}$ was reduced to MnO while $\hbox {Ta}_{2}\hbox {O}_{5}$ remained unchanged. (**C**) and (**D**) The red dot represents the reduction conditions for Step 2 at 1250$^{\circ }$C. In this step, MnO from Step 1 remained unreacted, whereas $\hbox {Ta}_{2}\hbox {O}_{5}$ was reduced to TaC in the form of agglomerates. (**E**) The phase diagram shows the final step for reducing MnO to $\hbox {Mn}_{7}\hbox {C}_{3}$, with the temperature maintained at 1350$^{\circ }$C.

Prior to running the microwave experiment, it was necessary to determine the temperature and pressure of the processing steps. The number of processing steps depends on the composition of the elements to be extracted and their respective oxide forms. In this study, we focus on refining Ta and Mn. We determined that three processing steps are required to recover these two elements. To aid in determining the correct processing steps, a thermodynamic analysis of the overall experiments was based on two thermodynamic graphical tools that are both quantitative and qualitative: the Ellingham diagram (ED) shown in Fig. 3 and the Phase Diagram (PD) shown in Fig. 4.

The ED is based on NIST-JANAF Thermochemical Tables, while the PD is based on CALculation of PHAse Diagrams (CALPHAD) thermodynamic calculations. In this research, we elected to use the FactSage CALPHAD software and the built-in thermodynamic databases for oxides (FToxide) and pure substances (FactPS). It should be noted that many of these databases are based on the NIST-JANAF tables, especially for pure substances. The approach involves using the ED to determine at what temperature the reduction process is thermodynamically favorable. Then, the PD is used to determine what equilibrium phases will form at these temperatures and the desired pressure to form a stable phase.

The ED for our process compositions is shown in Fig. 3. There are some underlying assumptions in the ED that are beyond the scope of this paper, but to highlight a major assumption: at high temperatures, the change in entropy dominates the change in Gibbs free energy of the reaction. Therefore, the latent heat or changes in enthalpy are not captured when a phase change occurs. However, this accounting is captured in the CALPHAD model, so we use the ED first and then the PD. Looking at the ED, the more negative the value, the more stable the product^66^. This means oxides like calcium oxides and silicon oxides (not shown in Fig. 3) are good flux materials because they have more negative values on the ED. It should be reiterated that the lines on the ED represent the Gibbs energy of the reaction, not the formation energy of the product. The y-intercept of the ED is called the enthalpy of the reaction ($\Delta H_f^o$) at a standard state of 298 K and 1 atm. The real utility of the ED is to pinpoint inflection points or points where different lines intersect. At these points, there is a change in the stability of the reactions.

In this study, we utilized carbon as the reducing agent. The real utility of using carbon is visualized in the ED by the green lines, either having no slope (in the case of $\hbox {CO}_{2}$) or a negatively sloping line (in the case of CO). Carbon reacting with oxygen (from oxides) will form either carbon monoxide (CO) or carbon dioxide ($\hbox {CO}_{2}$). The temperature where CO becomes more thermodynamically stable can be seen on the ED where the 2C + $\hbox {O}_{2}$ = 2CO line intersects the $\hbox {C} + \hbox {O}_{2} = \hbox {CO}_{2}$ line. This happens around 700$^{\circ }$C, where the CO product becomes more stable than $\hbox {CO}_{2}$. Moreover, the CO line is negatively sloped, which makes it an even better reducing agent. This is one reason why CO is the main reducing agent used in basic steelmaking.

We differentiate our process from the steelmaking process in that we rely on solid-solid reduction with the additional benefit from CO. In steelmaking, CO is the main reducing agent, and solid-solid accounts for a small fraction. There are many details that have been optimized over the last century, related to surface tension, flowability, and exothermic heat generation. The process presented here relies on solid-solid reduction as a result of the high melting temperature of Ta. The reduction process is facilitated by dual reduction pathways enabled by microwave-assisted heating. The first, direct reduction, occurs through the interaction of carbon with microwave energy, which allows efficient coupling and direct solid-solid contact between carbon and metal oxide. This promotes solid-state diffusion and accelerates the reduction process. The second, indirect reduction, involves carbon monoxide (CO), produced during the process, which acts as a reducing agent in a gas-solid interaction with the metal oxide.

By utilizing both solid-state and gas-phase reduction, our process enhances the overall efficiency of metal recovery, improving both the reduction kinetics and purity of the recovered metals. The combination of these two mechanisms, along with the benefits of microwave heating, underscores the potential of this approach for high-purity metal extraction, particularly for high-melting-point metals like tantalum.

Looking back at the ED figure (Fig. 3), the same approach can be used to determine the reduction temperature for Ta and Mn oxide. The intercept point of the metal oxide reaction ($\hbox {M} + \hbox {O}_{2} = \hbox {MOx}$) indicates that any temperature beyond this point makes $\hbox {CO}_{2}$ or CO more stable than MOx, meaning it can be reduced by carbon. The lines of interest for this study are $\frac{4}{5}{Ta} + \hbox {O}_{2} = \frac{2}{5}\hbox {Ta}_{2}\hbox {O}_{5}$ and $\hbox {Mn} + \hbox {O}_{2} = \hbox {MnO}_{2}$. Another important concept of the ED is that the difference between the equations $2\hbox {C} + \hbox {O}_{2} = 2\hbox {CO}$ and $\frac{4}{5}{Ta} + \hbox {O}_{2} = \frac{2}{5}\hbox {Ta}_{2}\hbox {O}_{5}$ results in $\frac{4}{5}{Ta} + 2CO = 2C + \frac{2}{5}\hbox {Ta}_{2}\hbox {O}_{5}$. This difference is visualized as the vertical arrows in Fig. 3 and the magnitude quantifies the Gibbs energy of the reaction. The reduction temperatures were determined to be 700$^{\circ }$C, 1250$^{\circ }$C, and 1350$^{\circ }$C for our proposed three-step process.

Once the reduction temperatures are determined from the ED, the PD provides the exact pressure to operate at and the resulting equilibrium phases. The experiment was divided into three steps. Each step was run in an inert atmosphere to prevent the oxidation of the materials. Because a vacuum was drawn and then back-purged with argon, the partial pressure of oxygen remained between 10$^{-3}$ and 10$^{-5}$ ${\rm atm\,P}_{O2}$. The temperatures of the steps were 700$^{\circ }$C, 1250$^{\circ }$C, and 1350$^{\circ }$C. As shown by a red dot in Fig. 4A–D, the pressure for the first step was held at 1 atm, the second step the pressure was 1.8 atm, while it was lowered to 0.3 atm in the third step (Fig. 4E). The vacuum atmosphere was first created inside the tube that held the sample, followed by a back-purge of argon gas to maintain an inert atmosphere. Since the sample comprised oxides of Ta and Mn, it was of utmost importance to maintain the pressure inside the reactor. Failure to do so resulted in a mixture of compounds that could not be separated. This was discovered early in the experiments with the formation of tantalite (MnTa$_2$O$_6$), which, incidentally, is the ore phase from which Ta is typically mined. The aim of staging the process was to first reduce $\hbox {MnO}_{2}$ to a lower oxide (MnO) in the first step, followed by the reduction of $\hbox {Ta}_{2}\hbox {O}_{5}$ into TaC in the second step, and the final reduction of MnO to $\hbox {Mn}_{7}\hbox {C}_{3}$ in the last step.

Based on the ED (Fig. 3) and PD (Fig. 4), the path for the reduction of $\hbox {MnO}_{2}$ to MnO (a$\rightarrow$ a” in Fig. 3) is shown as:

10 $$\begin{aligned} {\rm MnO}_2+ {\rm C} \rightarrow {\rm MnO} + {\rm CO}. \end{aligned}$$

Likewise, the path for the reduction of $\hbox {Ta}_{2}\hbox {O}_{5}$ to TaC (b$\rightarrow$ b” in Fig. 3) can be described as:

11 $$\begin{aligned} {\rm Ta}_2{\rm O}_5+ 2{\rm C} \rightarrow \frac{5}{2}{\rm O}_2+2{\rm TaC}. \end{aligned}$$

Finally, the final path for the reduction of MnO to $\hbox {Mn}_{7}\hbox {C}_{3}$ (c$\rightarrow$ c’ in Fig. 3) can formulated as the following:

12 $$\begin{aligned} \frac{7}{3}{\rm MnO}+ {\rm C} \rightarrow \frac{1}{3}{\rm Mn}_7{\rm C}_3+ \frac{7}{6}{\rm O}_2. \end{aligned}$$

### Finalized procedure

**Fig. 5:**
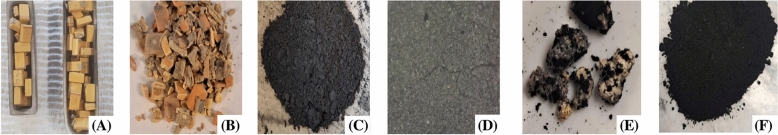
Photographs of the overall process at each stage. (**A**) As obtained TCs, (**B**) TCs obtained after heating, (**C**) Ground TCs and carbon, (**D**) mixture obtained after the first MW process step, (**E**) TaC sponge material obtained after the second MW process step, and (**F**) Remaining powder after third MW process step.

**Fig. 6:**
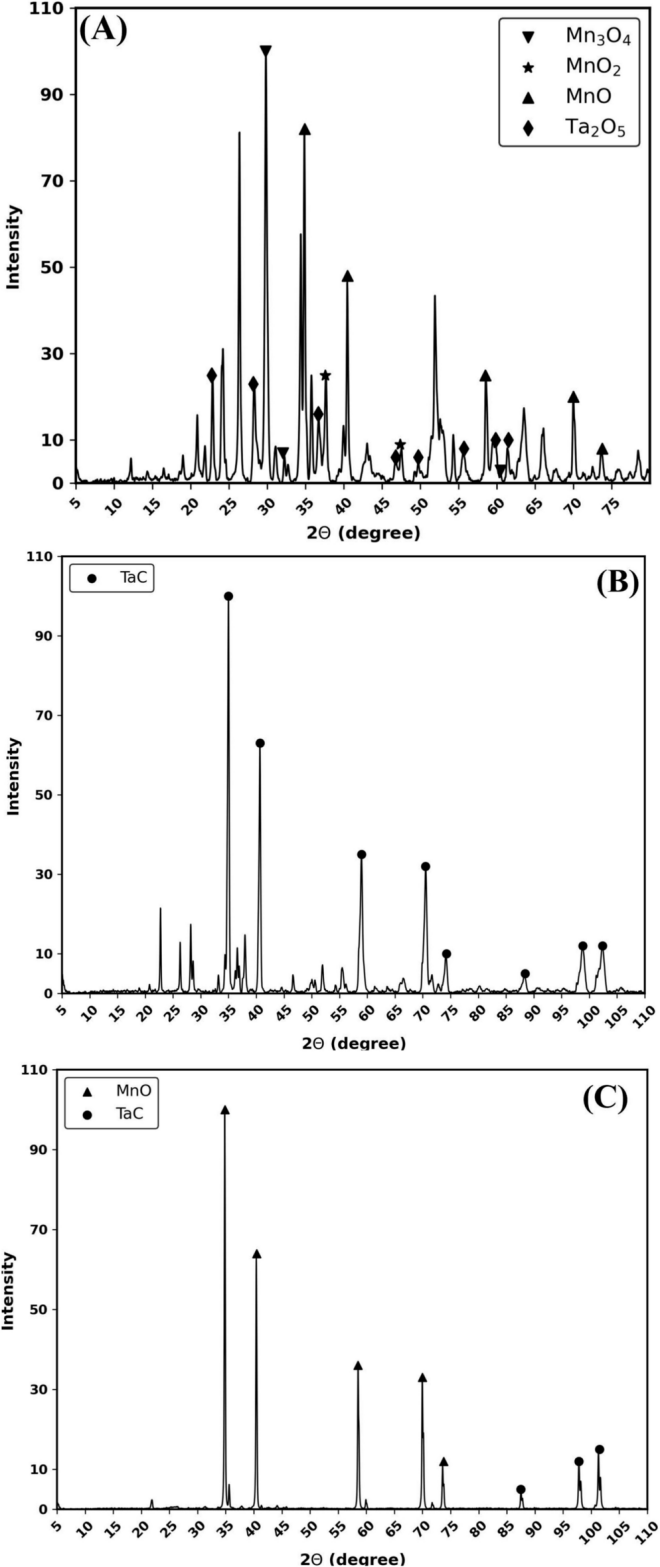
XRD analysis of the product after each step. (**A**) Step 1: $\hbox {MnO}_{2}$ was converted to a lower oxide form (MnO), while $\hbox {Ta}_{2}\hbox {O}_{5}$ remained unreacted, as expected. (**B**) Step 2: A 97% purity TaC agglomerate was physically separated. (**C**) Step 3: The lower oxide forms of $\hbox {MnO}_{2}$ were fully converted to MnO in the final step. Traces of TaC were observed due to limitations in the physical separation technique used after Step 2.

The experiments were performed following the methodology described in the Methods section. The overall experimental process and results obtained are illustrated in Fig. 5 with photographs of products for each step. For Step 1, 1.0 *g* of finely crushed TCs were mixed with 0.2 *g* of carbon black (lampblack) powder (lot no. S22E047, Alfa Aesar 39724, Thermo Fisher Scientific) and roll-milled for 10 min to form a homogeneous mixture. After the completion of Step 1 (Fig. 4A,B), $\hbox {MnO}_{2}$ was reduced to MnO. The XRD for the powder obtained after the first step run is shown in Fig. 6A. It was found that the majority of the $\hbox {MnO}_{2}$ was converted to MnO, with small traces of $\hbox {Mn}_{3}\hbox {O}_{4}$ and $\hbox {MnO}_{2}$ also observed. $\hbox {Ta}_{2}\hbox {O}_{5}$ remained as is after Step 1, as expected.

Step 2 (Fig. 4C,D) was executed after adding 0.1 *g* of additional carbon to the sample obtained from Step 1. As a result of Step 2, small diameter TaC sponge material was obtained as seen in Fig. 5E. The 0.44 *g* of TaC material obtained after Step 2 was physically separated (diameter of material approximately 1 *mm* as shown in Fig. 5E) and characterized using XRD provided in Fig. 6B. XRD confirmed the phase of TaC, showing distinct TaC diffraction peaks. The purity of the material was then evaluated using quantitative ICP-MS, which revealed a Ta purity of 97%. The formation of TaC instead of the metallic phase is due to phase stability at atmospheric conditions. Metallic Ta readily oxidizes in an oxygen atmosphere to tantalum pentaoxide. This is exacerbated for fine particles with high areal density. The formation of TaC provides a stable phase that can be stockpiled and handled in the open atmosphere.

Finally, after the physical separation of large-diameter ($>0.5~{\rm mm}$) TaC sponge material, the remaining powder was further processed in Step 3 (Fig. 4E). However, the resulting products of Step 3 were not the expected carbide material based on the PD. Instead, a lower oxide, MnO, was present, as in Step 2. This was surprising, given that manganese carbide was anticipated from the PD.

Further investigation revealed that $\hbox {Mn}_{7}\hbox {C}_{3}$ is not stable above 1300$^{\circ }$C, which can be seen by the abrupt end of its line in the ED at 1100$^{\circ }$C. This discrepancy may be due to the thermodynamic database for Mn used in the CALPHAD model, which often treats Mn as an alloying element rather than a pure substance. Additional study is needed to confirm this hypothesis. Alternatively, this outcome could be the result of a localized heating effect of MnO under microwave irradiation, necessitating further investigation of the dielectric properties.

The phase and purity of Step 3 products were also examined using XRD and ICP-MS respectively. Unlike Step 2, where high purity TaC was observed, no significant amount of $\hbox {Mn}_{7}\hbox {C}_{3}$ was found, but the presence of MnO and TaC was confirmed. ICP-MS of the Step 3 product revealed a large amount of Ta still remaining, likely due to the limitation of the physical separation technique with only large agglomerates of TaC being removed after Step 2. In future studies, a more precise physical separation technique will be used to remove small particles of TaC. The remaining material after Step 3 took on a brownish color, synonymous with MnO, and was confirmed by the XRD results in Fig. 6C. It should be noted that XRD has limitations in differentiating between TaC and MnO, as they form similar crystal structures, with only slight differences in lattice spacing. Quantitative powder analysis (QPA) of the XRD pattern reported a higher concentration of MnO, while ICP-MS reported a higher concentration of Ta than Mn. The initial concentration is shown in Table 2 where Ta is over three times greater in concentration than Mn.

**Fig. 7:**
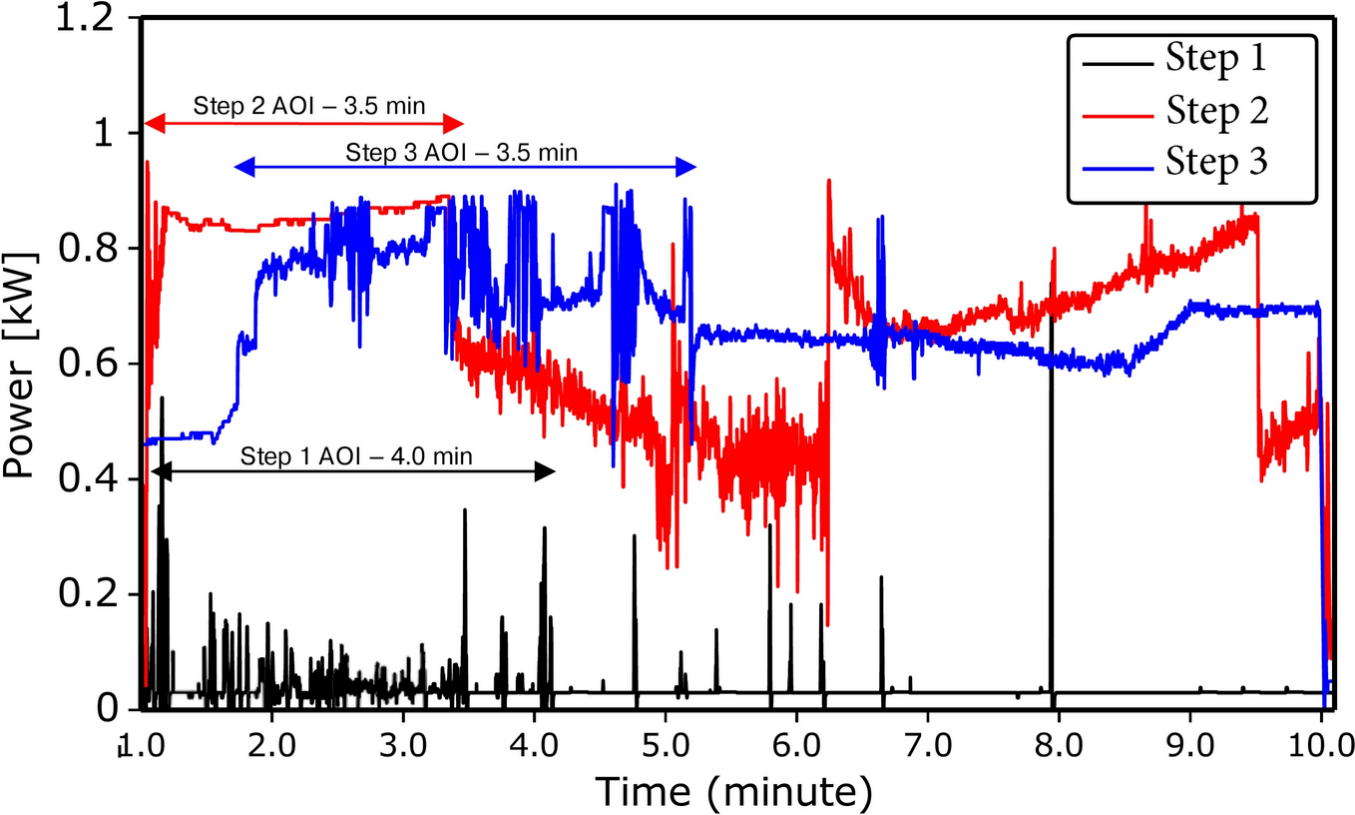
Power consumed during all three steps of the reduction process. The average power consumed in the first, second, and third steps were found to be 0.04, 0.63, and 0.64 kW, respectively. The power decreases for Step 2 and 3 after approximately 3.5 min was due to self-limiting of reaction.

In addition to the compositional analysis of each of the processing steps, the forward and reflected MW power for all three processes were monitored. Figure 7 summarizes these results. The power was monitored at the microwave source. The stub tuner and the sliding short were maintained in their positions for the duration of the run. The power of the MW was varied by the LabVIEW PID controller to maintain a set point temperature. The microwave source had a continuous power output and was not pulsed. The average power for each step was 0.04 kW, 0.63 kW, and 0.64 kW, respectively. These are low powers to achieve temperatures in the thousands of degrees, attributable to the direct deposition of power rather than heating the whole unit, as in conventional heating. Additionally, some exothermic chemical energy derived from the reaction contributed to heating the sample, allowing quick on-and-off operation. Moreover, utilizing the chemical energy, as in basic steel making, means the microwave unit only needs to initiate and sustain the reaction rather than supply all the reaction energy.

Further investigation of power utilization in Fig. 7 reveals that the reaction stops after a certain point, indicating the end of the reduction for that step. This illustrates that the reaction, being a solid-solid interaction and heated locally, is self-limiting. For Steps 2 and 3, there is a noted decrease in absorbed power at approximately 3.5 min as a result of the reaction stopping and a resulting phase change. Therefore, there is evidence that microwave reduction is quick and self-limiting, providing potential for scaling the technology to larger batch reactors.

## Conclusion

End-of-life tantalum capacitors (TCs) containing 42% Ta were successfully processed to yield high-purity tantalum using a staged microwave heating approach informed by Ellingham and phase diagrams. First, the capacitors underwent pyrolysis, which increased the tantalum purity from 42% to 70% while converting all organics (paints and polymers) to carbon. Subsequently, microwave-assisted carbothermal reduction was performed in three controlled stages, each set at specific temperatures and pressures. This selective, multi-step strategy allowed for efficient and self-limiting reactions, in contrast to conventional heating methods that often require higher energy inputs and longer processing times.

As a direct result, fine TaC sponge material (about 1 mm in diameter) containing 97% Ta was obtained and physically separated. Notably, the TaC phase offers enhanced stability in ambient environments and can be either stockpiled for later use or refined further to achieve ultra-high-purity tantalum suitable for microelectronic applications. Small traces of $\hbox {MnO}_{2}$ in the e-scrap were also reduced to lower oxides, which were then physically separated.

Overall, this study demonstrates that end-of-life TCs can serve as a viable secondary source of tantalum and that microwave heating, particularly when coupled with both direct and indirect carbothermal reduction, presents a rapid and energy-efficient route to recovering high-value metals from heterogeneous e-scrap. Beyond its relevance to tantalum, the approach can be extended to a wide range of materials, providing a new perspective on microwave-assisted extraction and refining in the context of sustainable resource recovery.

## Supplementary Information

Supplementary Information.

## Data Availability

Data including XRD data, Ellingham diagram, Phase Diagrams, and Multiphysics COMSOL files will be made available on GitHub at https://github.com/Dr-Musho-Research-Group/.
