# Supplementary material for: Microwave-assisted recycling of tantalum and manganese from end-of-life tantalum capacitors
Source: Sci Rep. 2025 Apr 11;15:12366. doi: 10.1038/s41598-025-96574-7 (PMC11986002; doi:10.1038/s41598-025-96574-7)
Supplement: Supplementary file 1 — Supplementary Information. [file 41598_2025_96574_MOESM1_ESM.pdf]

Supplemental Information

A COMSOL Multiphysics model was constructed, as shown in Figure S1. COMSOL Multiphysics version 6.1 was used for this analysis. COMSOL is a finite-element-based solver designed to couple multiple physical phenomena. This problem is a good candidate for a multiphysics simulation because the dielectric properties strongly depend on temperature. For dielectric materials, the imaginary part of the dielectric constant often increases with temperature. For metals, the opposite is true because the imaginary part is proportional to the electrical conductivity of the material. However, for dielectric materials, absorption can increase exponentially with temperature. It is essential to capture this effect to model the realistic response of microwave heating. Moreover, this is crucial for using the model to help match the system.

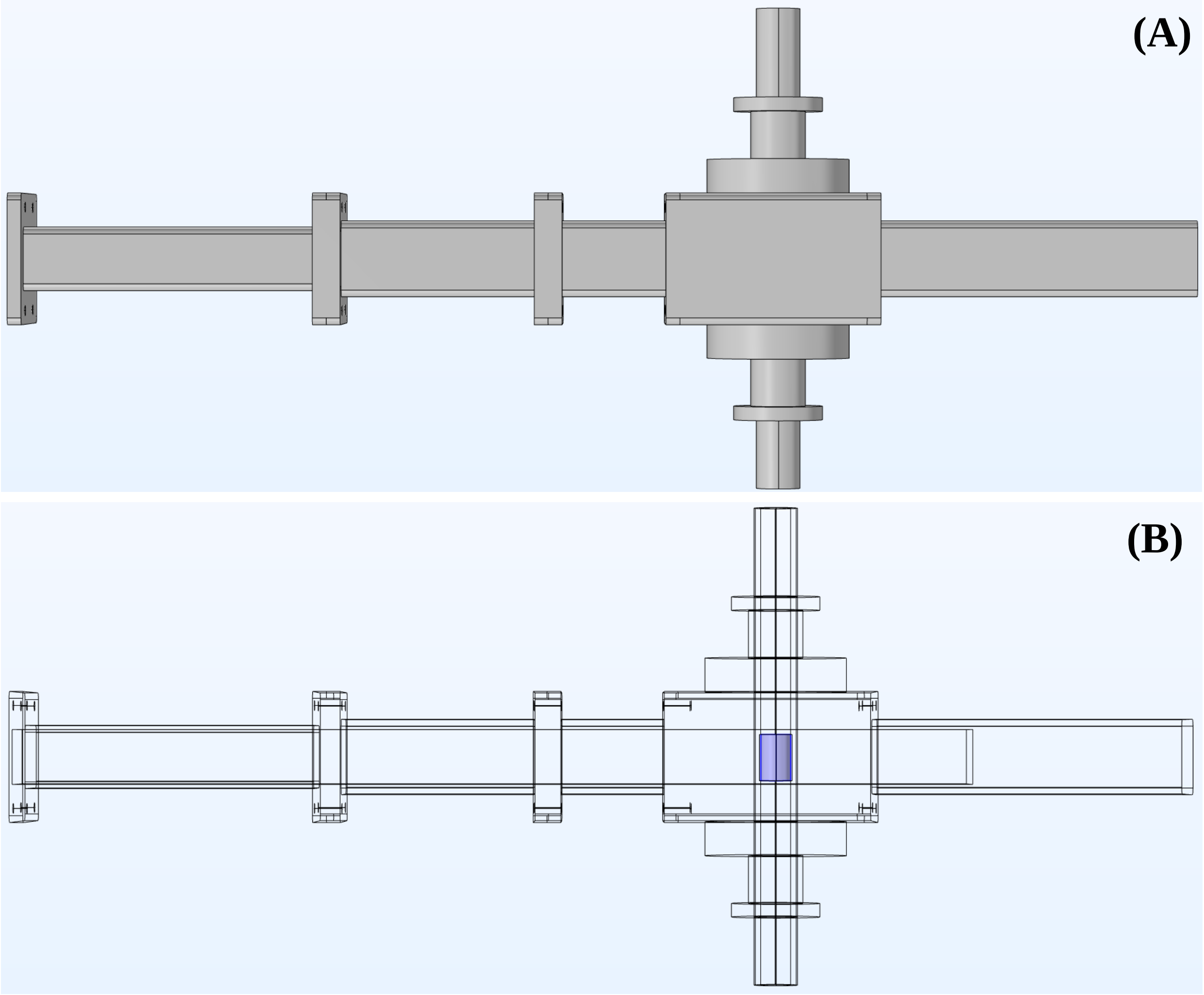

Figure S1: CAD model of the microwave reactor used during the experiment. (A) External geometry of the waveguide. (B) Wireframe view of the microwave reactor with the sample highlighted in blue.

The COMSOL model couples RF heating with heat transfer as seen inf Figure S2. The RF COMSOL module is used to solve Maxwell's equations in the frequency domain; hence, the RF model is a Finite

Element Frequency Domain (FEFD) solution. This approach offers several advantages over others. The finite element method is well-suited for complex geometries because an unstructured mesh with high-order elements can be used to accurately capture detailed geometry. In COMSOL, the finite element solution is obtained using an implicit scheme, which does not depend on a strict stability criterion. In principle, any time step can be chosen for the heat transfer solver, and the solution will still converge. However, caution must be taken to avoid missing any relevant physics in the coupling process. Since Maxwell's equations are solved in the frequency domain (rather than the time domain), waves are assumed sinusoidal, and material properties cannot vary with frequency. This assumption is valid because the source operates at a fixed frequency (2.45 GHz) and the material is assumed to be non-dispersive.

The heat transfer model is solved by coupling the RF module with the Heat Transfer (HT) module through a multiphysics interface. The coupling is two-way: the volumetric heat generation (Q_h) computed by the RF module is provided to a transient heat conduction equation, and the resulting temperature is passed back to the RF model, where the temperature-dependent dielectric properties are evaluated. For each time step, determined by the transient heat transfer solver, the two-way coupling solution is iterated until a convergence criterion is met, and then proceeds to the next time step until the final simulation time is reached.

It should be noted that a crucial boundary condition for the transient heat conduction model is radiation. Because temperatures above 500 °C are reached, radiative heat transfer dominates the thermal losses from the sample to the surroundings. Since the applicator walls are water-cooled, we employ a radiation-to-surroundings boundary condition that neglects any reflected power. Essentially, the sample is treated as a diffuse gray body with a view factor of unity.

As shown in Figure S2A, the electric field is concentrated near the sharp edge of the sample. This positioning maximizes the volumetric heat flux into the sample. The corresponding temperature contour plot (Figure S2B) reveals that the highest temperature occurs at the leading edge of the sample, which is closest to the microwave source. While surface electric fields play a role, the electric field distribution within the sample is often the most critical factor for heating. Because this material is a dielectric, it has a complex index of refraction characterized by a nonzero extinction coefficient, resulting in significant attenuation (lower transmissivity). In other words, although the sample behaves largely opaquely, it does not occupy the full cross-section of the waveguide, allowing part of the wave to bypass the sample without being fully absorbed. Under this opaque assumption, any energy not reflected is absorbed by the material. Consequently, in Figure S2A, the electric field attenuates quickly inside the sample, and the unabsorbed portion is reflected, leading to high surface electric fields around the sample.

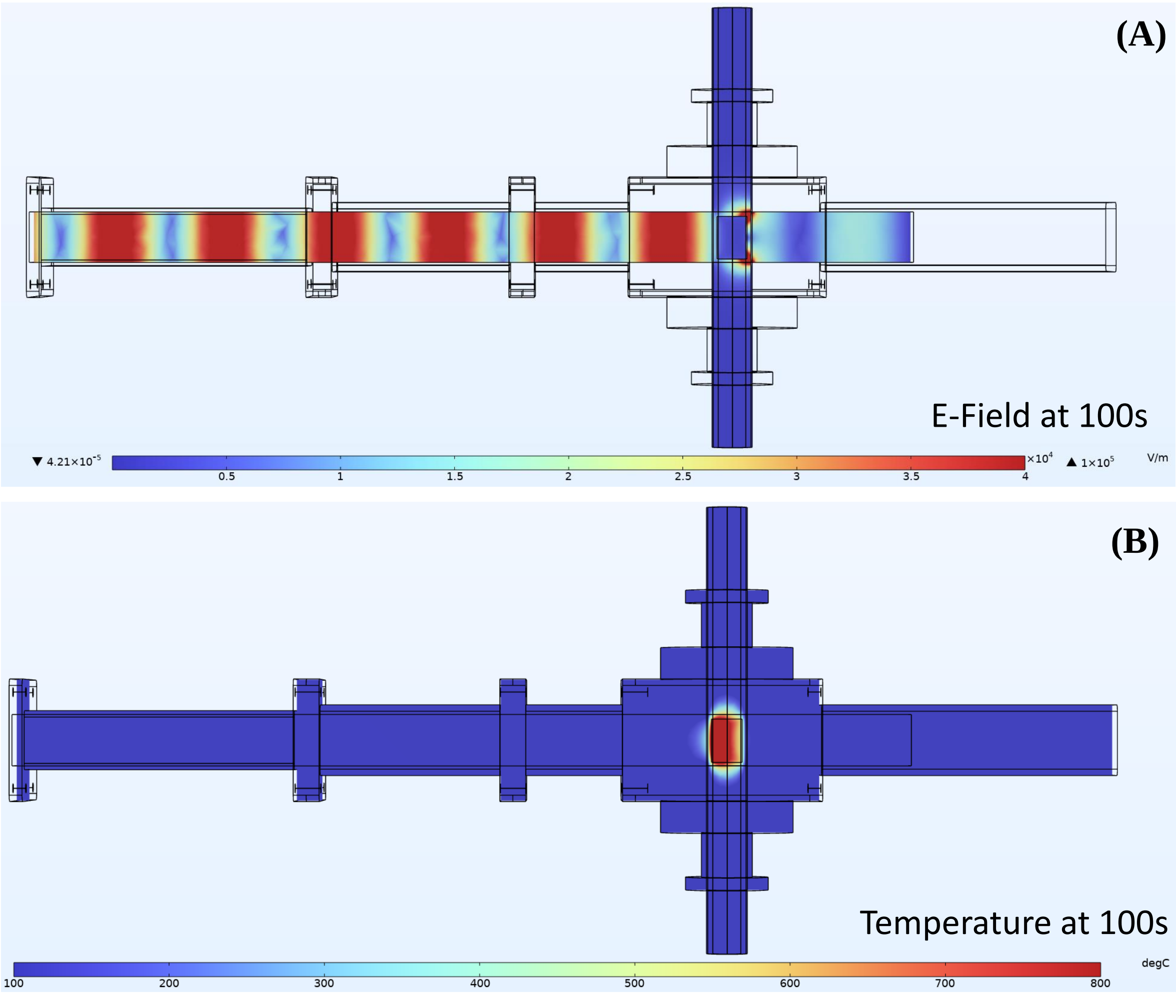

Figure S2: COMSOL Multiphysics simulation results. (A) Electric field within the waveguide and sample at 100 s. Note the high electric field around the sample as a result of its conductive nature. (B) Temperature profile illustrating the high temperature of the sample reached at 100 s.
